# Supplementary material for: Whole-Genome Analysis of Acinetobacter baumannii Strain AB43 Containing a Type I-Fb CRISPR-Cas System: Insights into the Relationship with Drug Resistance
Source: Molecules. 2022 Sep 2;27(17):5665. doi: 10.3390/molecules27175665 (PMC9458022; doi:10.3390/molecules27175665)
Supplement: Supplementary file 1 [file molecules-27-05665-s001.zip › molecules-1829660-supplementary.pdf]

Table S1. Comparisons of the chromosome and plasmid of *Acinetobacter baumannii* strain AB43 with *A. baumannii* strain ATCC 19606 and *A. baumannii* strain ATCC 17978.

Strains	Chromosome			Plasmid		
	Size (bp)	GC (%)	Number of Genes	Size (bp)	GC (%)	Number of Genes
AB 43	3854806	39.1	3751	104309	40	112
ATCC 19606	3980901	39.2	3807	18598	34.6	28
ATCC 17978	4006609	39.0	3848	16502	34.6	20

Table S2. Spacer sequences and their matched genes using CRISPR Target (http://crispr.otago.ac.nz/CRISPRTarget/crispr_analysis.html) based on the Refseq-plasmid and Genebank-phage database.

Space No.	Space Sequence	Length	Similarity(%)	Matched to (matched range)	Protein ID
15	TAGTCCCGAAA TTTCAGTAGGT GGCTCATAGC	32	97	Acinetobacter junii strain YR7 plasmid pNDM-YR7 (NZ_CP059559.1) **DUF1173 family protein** position: 42207-42238	WP_015060702.1
20	GTTACGTCTAC GGTACGTGGTT AGCATGATTT	32	100	Acinetobacter lwoffii strain SU1904 plasmid pSU1904NDM(NZ_LC537594.1) **VirB4** position: 26098-26067	WP_015060708.1
21	CCTGCCGTTAA TTCACCGCTTG GGTGATAGAA	32	100	Acinetobacter junii strain YR7 plasmid pNDM-YR7 (NZ_CP059559.1) **VirB4** position: 27072-27041	WP_015060708.1
22	GCAGGGAAAA GTATAGCTTTA GATATATCCCA	32	100	Acinetobacter schindleri strain ACE plasmid p2AsACE, (NZ_CP015617.1) hypothetical protein position: 37517-37548	WP_076755199.1
24	TTGCTCTAAGC ATTCCGAAGCA AGGAAATGTG	32	91	Acinetobacter phage 4316, complete genome.(OM334891) **DUF2800 domain-containing protein** position: 26905-26936	UKM17106.1
26	TGATCTTTGCA ACCAAGCATTC AACGAATCCA	32	94	Alcaligenes phage Piluca.(MZ326864) hypothetical protein position: 15682-15653	QYW02374.1
27	AATTTCATGCTT ATGTGCTAAAT TCATATTTCA	32	97	Alcaligenes phage Piluca.(MZ326864) **HNH endonuclease** position: 14627-14595,	QYW02373.1
30	GTGTTGTAATTT CGCTTGTATTGA TCATTGCC	32	100	Acinetobacter junii strain YR7 plasmid pNDM-YR7 (NZ_CP059559.1)	WP_004999358.1

				hypothetical protein position: 29117-29086	
31	ACATACCCCAT ATAACGGTTAT TGTAGTTGCT	32	94	Acinetobacter schindleri strain ACE plasmid p2AsACE, (NZ_CP015617.1) hypothetical protein position: 37365-37395	WP_076755199.1
35	AAGTGAAAAA AGCTGACAGCA AAATAAACTCA	32	100	Acinetobacter phage fLi-Aba02.(MT344104) **integrase** position: 21870-21839,	QJT69935.1
41	TTAAGCAATCGC AAAACATTGCAG CTTTAGACC	33	100	MZ326864.1 Alcaligenes phage Piluca.(MZ326864) **pilot protein** position: 32904-32936	QYW02397.1
46	AAACTGCATAGA TGACCATTAGAG ACATCCCC	32	100	Acinetobacter baumannii strain Ab-D10a-a plasmid pAb-D10a-a_4(NZ_CP051873.1) position: 4219-4250	—
47	GTAGTGGGGAC ATTGTGTACGCT CATGATGTT	32	100	Acinetobacter baumannii strain DETAB-E227 plasmid pDETAB5 (NZ_CP072528.1) **recombinase family protein** position: 76626-76657	WP_004993315.1
48	TCATGCCAGTTG CAAAGTCCAAGC CCACTGCT	32	100	Acinetobacter schindleri strain ACE plasmid p2AsACE (NZ_CP015617.1) hypothetical protein position: 37602-37633	WP_076755199.1
54	ATGATCTACCCT TTGAGCTAGATA AAACTTTT	32	100	Acinetobacter junii strain YR7 plasmid pNDM-YR7 (NZ_CP059559.1) **recombinase family protein** position: 32966-32997	WP_004993315.1
55	TTCACGTGGTAC GCGCTGTAAGCC TGTTTGTT	32	94	Alcaligenes phage Piluca.(MZ326864) hypothetical protein position: 17743-17714	QYW02378.1
64	ACTTTAAGCGCA AACGAACAGCA TCAAGATCA	32	91	Acinetobacter phage AM106 chromosome linear.(MH115576) **putative main tail protein** position: 30457-30426	AWD93169.1
65	TGTTTCATCTGT CTTGATAGCTTC CCCCATAA	32	100	Acinetobacter schindleri strain ACE plasmid p2AsACE, (NZ_CP015617.1) **DUF1173 family protein** position: 38699-38730	WP_076755202.1
66	CACTACGGACAT AATCGAACATTT CCTGAAGT	32	100	Acinetobacter junii strain YR7 plasmid pNDM-YR7 (NZ_CP059559.1) **recombinase family protein** position: 33067-33036	WP_004993315.1
74	GTCAGTATTACT CCACACTTTTTA TATTTCAC	32	94	Acinetobacter towneri strain GX5 plasmid pGX5 (NZ_CP071769.1) **Y-family DNA polymerase** position: 122055-122024	WP_000100479.1

87	TGCATCGAAGTC ATAGAACGCTTT GAGTTTCCA	33	94	Acinetobacter phage 4316, complete genome.(OM334891) **DNA polymerase I** position: 22470-22502	UKM17101.1
90	GCAAGTGTCACC TTTAAGTGCACG GAAGAAAT	32	100	Alcaligenes phage Piluca.(MZ326864) hypothetical protein position: 14277-14308	QYW02372.1
92	TTGGTTGTTTAG TAAGTTCAAAGC CCGTTGTA	32	97	Acinetobacter phage Ab105-2phideltaCI404ad.(MZ514874) **glycosylhydrolase 108** position: 51123-51092,	QZI85302.1
93	CAATGGATTTAA AGATTTCAACCG CTTGGGAG	32	97	Acinetobacter phage fLi-Aba02.(MT344104) **integrase** position: 22182-22152	QJT69935.1
95	TCACTTCTCTTA ATTAGTGCTGAA GAATCAAA	32	100	Acinetobacter baumannii strain D4 plasmid pD4, complete sequence(NZ_CP048851.1) hypothetical protein position: 26274-26243	WP_000875973.1
96	CTGAATAAGTGA GATGTCTCTATG AGTTCAATT	33	100	Acinetobacter baumannii strain KSK20 plasmid p1KSK20(NZ_CP072301.1) hypothetical protein position: 205874-205842	WP_002015155.1 WP_000961970.1
97	ATTGAATTAATA CGGCTTACTGCA ATTGGTTG	32	91	Alcaligenes phage Piluca.(MZ326864) **tail protein** position: 32523-32492	QYW02396.1
103	AACGTGAACAAT TGTTTTAGCTTT GAACATTA	32	91	Acinetobacter pittii strain A1254 plasmid pA1254_2 (NZ_CP049808.1) **DNA topoisomerase** position: 15549-15580	WP_167564493.1

Table S3. Predicted prophage regions in *Acinetobacter baumannii* strain AB43 using PHAge Search Tool (PHAST) web server (http://phast.wishartlab.com/).

Region	Completeness	Most common phage	Region length (bp)	Region position	GC
1	Intact	Acinet_Bphi_B1251, Acinet_LZ35	52250	2156093–42208342	38.45%
2	Incomplete	Acinet_Acj61, Pseudo_Pq0	31033	1265100–1296132	40.06%
3	Incomplete	Acinet_vB_AbaS_TRS1, Acinet_LZ35, Acinet_Bphi_B1251	16326	2484593–2500918	38.47%
4	Incomplete	Acinet_Bphi_B1251	5786	2547454–2553239	41.38%
5	Incomplete	Salmon_SJ46, Acinet_Bphi_B1251	4730	33397–38126	35.79%
6	Incomplete	Entero_BP_4795, Entero_VT2phi_272	8610	42102–50711	37.84%
7	Questionable	Acinet_Bphi_B1251	50605	1214593–1265197	39.62%
8	Questionable	Burkho_phi1026b	28519	2855501–2884019	40.82%
9	Questionable	Burkho_phiE12_2	13486	88779–102264	39.74%

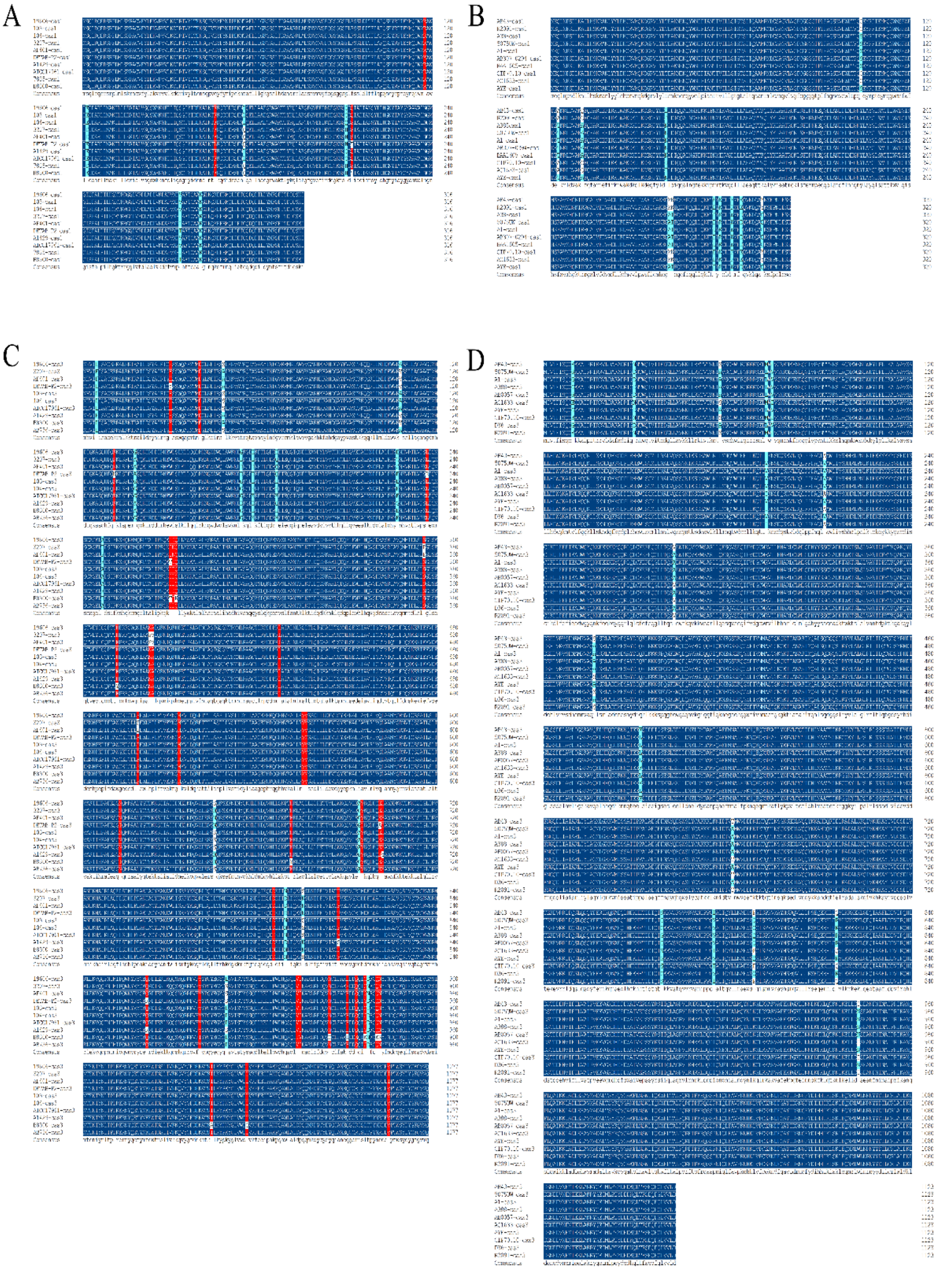

Figure S1. A: Alignment of Cas1 amino acid sequences in the *Acinetobacter baumannii* Type I-Fb CRISPR-Cas system–containing strain**. B:** Alignment of Cas1 amino acid sequences in the *A. baumannii* Type I-Fa CRISPR-Cas system–containing strain. **C:** Alignment of Cas3 amino acid sequences in the *A. baumannii* Type I-Fb CRISPR-Cas system–containing strain**. D:** Alignment of Cas1 amino acid sequences in the *A. baumannii* Type I-Fa CRISPR-Cas system–containing strain.
